# Advancing Aortic Dissection Imaging: First Clinical Experience of Photon-Counting CT with Ultra-Fast Spectral Imaging

**DOI:** 10.3390/diagnostics15202655

**Published:** 2025-10-21

**Authors:** Daniel Dillinger, Maria Weiss, Hanns L. Kaatsch, Christian Bauer, Achim Hagen, Matthias F. Froelich, Stephan Waldeck, Daniel Overhoff

**Affiliations:** 1 Department of Vascular and Endovascular Surgery, Federal Armed Services Hospital Koblenz, 56072 Koblenz, Germany; 2 Department of Radiology and Neuroradiology, Federal Armed Services Hospital Koblenz, 56072 Koblenz, Germany; 3 Department of Radiology and Nuclear Medicine, University Medical Center Mannheim, 68167 Manheim, Germany; 4 Institute of Neuroradiology, University Medical Centre, Johannes Gutenberg University Mainz, 55131 Mainz, Germany

**Keywords:** aortic dissection, photon-counting detector CT

## Abstract

**Background**: Computed tomography (CT) is the standard of reference for diagnosis and follow-up in aortic dissection (AD). Localizing the entry and identifying false and true lumen are as important as differing post-treatment changes from contrast media extravasations. Photon-counting detector CT (PCDCT) allows for virtual monoenergetic (VME) reconstructions, which can augment contrast media effects on lower energy levels, and for virtual non-contrast (VNC) reconstructions. The aim of this study was to analyze the influence of VME reconstructions on contrast media effects in different dissection compartments as well as compare true and VNC series in AD patients. **Methods**: We retrospectively analyzed PCDCT datasets from 28 patients with aortic dissections, with different dissection types and different treatment statuses. Attenuation and standard deviation values of the ascending and descending aorta, as well as CT values of the false lumen, were measured. These measurements were obtained from VME images at energy levels ranging from 40 to 190 keV in 10 keV increments, as well as from non-contrast (NC) and VNC reconstructions. The signal-to-noise ratio (SNR) was calculated. Additionally, subjective values for dissection assessability and native aspects of the images were acquired for different reconstructions. **Results**: CT values decreased with higher energy levels in VME imaging. Ascending aorta showed higher attenuation values than descending aorta, which was higher than false lumen (e.g., at 70 keV ascending 357 [310; 419] HU, descending 346 [305; 401] HU and false lumen 298 [248; 363] HU). These differences increased on lower VME reconstructions with statistical significance for the comparisons of ascending and descending aorta with the false lumen on all energy levels. In line with this, SNR showed highest values for ascending aorta compared to descending aorta and false lumen on all energy levels. For NC comparisons, VNC and VME at 190 keV reconstructions showed higher CT values than NC reconstructions (e.g., overall data NC 48 [42; 55] HU, VNC 66 [57; 73] HU, 190 keV 97 [89; 105] HU). Subjective ratings were worse with VNC than with NC images. **Conclusions**: VME reconstructions on lower energy levels can be helpful in differentiating between true and false lumen in aortic dissections.

## 1. Introduction

Aortic dissection (AD) is a life-threatening condition characterized by a tear in the aortic intima, associated with high morbidity and a mortality rate of about 50% within the first 48 h [1]. Mortality rates increase if not treated by 1% per hour, incidence is described between 2.5 and 15 per 100,000 per year [2]. Depending on the location of the entry, aortic dissections were primarily classified as “Stanford A” (dissections affecting the ascending aorta), “Stanford B” (a dissection only affecting the descending aorta), that classification was later extended to “Non A Non B” (aortic arch is involved) dissections. In 2020, Sievers et al. proposed the TEM classification which included the Stanford type (“T”), the entry location (“E”) and malperfusion (“M”) [3]. That classification was also mentioned in the current guidelines of the European Association for Cardio-thoracic Surgery [4] and prognosis depends on the entry location [3]. Computed tomography (CT) is central to the diagnosis of aortic dissection and is widely used to guide therapeutic decision-making. ECG-gated non-contrast- and contrast-enhanced multiphase CT scans are not only standard of reference in primarily diagnosing AD with sensitivity and specificity of 100% and 98–99% [5,6] but are also useful in follow-up examinations to rule out complications (e.g., persistent perfusion of false lumen, true lumen collapse or malperfusion of supraaortal, spinal or visceral arteries).

Photon-counting detector CT (PCDCT) became clinically available in the year 2021 and is already showed promising results regarding improved image quality and reduced radiation doses [7,8,9]. In addition to these given advantages, the inherent spectral data of PCDCT allows for virtual monoenergetic (VME) reconstructions at different energy levels. These proved helpful in increasing the present vascular contrast and showed (minor) effects on metal artifacts [10,11,12,13]. With decreasing energy levels (toward the k-edge of iodine), contrast media effects are increased due to an increased absorption [11]. PCDCT not only allows for the reconstruction of images at different energy levels, but the datasets can also be used for virtual non-contrast (VNC) reconstructions based on material deconstruction, which uses water and iodine as base materials [14].

Non-contrast (NC) series are important in AD imaging to help with the differentiation of hyperattenuated objects or areas (e.g., hyperdense felt sutures after ascending aortic replacement and contrast paravasations [6]). In addition, intramural hematoma can be identified in NC scans, but the acquisition of NC scans adds to the radiation dose which is applied to the patient. This (in the light of reoccurring examinations after AD therapy [6]) leads to the question of whether VNC or high energy VME reconstructions could replace a true NC scan. Also, low energy VME reconstructions might be helpful in detecting smaller contrast media leaks into the false lumen or other details (e.g., ulcer-like projections or intramural blood pools) in diagnostics of acute AD and CT scans after open surgery or endovascular therapy of AD. To the knowledge of the authors, no previous study has performed research on attenuation value differences in AD associated with the possible advantages of low keV reconstructions in differentiating true and false lumen nor in the comparison of high keV and VNC reconstructions for identifying intramural hematoma. Therefore, the aim of this study is to examine the effects of VME and VNC images on AD imaging in patients with acute AD and patients after treatment.

## 2. Materials and Methods

### 2.1. Study Design

We retrospectively analyzed all patients with aortic dissection—pre- and post-surgical or endovascular treatment—who underwent imaging using the dedicated aortic dissection protocol on the PCDCT in our institution from December 2021 to February 2024. Patients were excluded if the images were hampered by major artifacts or if either the non-enhanced or the spectral datasets were missing.

The local ethics committee approved the retrospective analysis of the data (2022-16314). Written consent was waived due to the retrospective design of this study.

### 2.2. Scanning Protocol

Patients were scanned on a PCDCT (NAEOTOM alpha Siemens Healthineers, Forchheim, Germany) with a standardized AD protocol. This specific protocol includes a non-enhanced CT scan, followed by a contrast-enhanced scan in the arterial phase of the chest and the abdomen. The second scan was triggered by placing a region of interest (ROI) in the ascending aorta, contrast media (Xenetix 350, containing 76.78 g/100 mL Iobitridol, Guerbet, Roissy, France) was injected by an injector (CT Motion XD8000, Ulrich Medical, Ulm, Germany) with a volume of 120 mL and a flow rate of 4 mL/s followed by a saline chaser of 50 mL at a flow rate of 4 mL/s. The scan started after reaching a threshold of 100 HU at 120 kV.

Both scans were ECG-gated and performed in QuantumPlus mode at 120 kV with tube current modulation (CARE Dose4D), a pitch of 3.2 and a collimation of 144 × 0.4 mm, image quality level 85 was used in these scans. The aortic root was scanned at the 40% RR-interval.

The acquired data were reconstructed with a Qr40 kernel and a slice thickness of 1 mm, the reconstruction interval was 1 mm.

Syngo.via (VB60, Siemens Healthineers, Forchheim, Germany) software was used to reconstruct the images on the different energy levels, including virtual non-contrast, VME images were reconstructed using Monoenergetic+ mode. CT dose index (CTDI) and dose length product (DLP) of NC- and contrast-enhanced scans were acquired.

### 2.3. Objective Analysis

**Figure 1:**
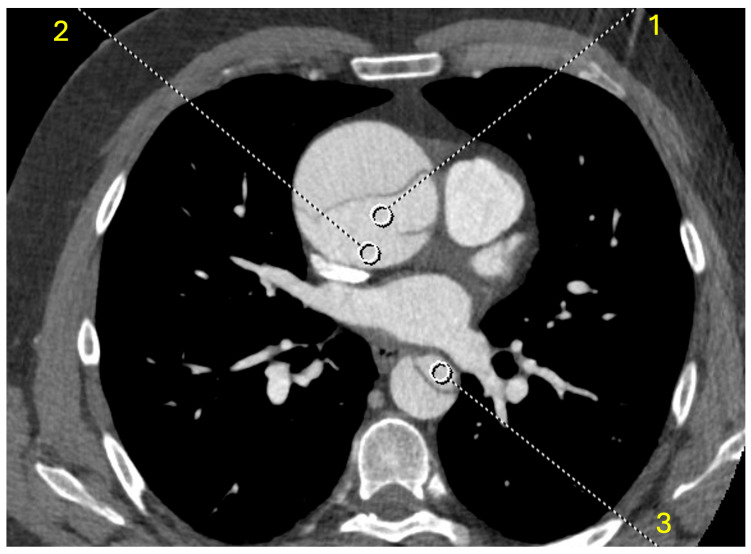
Demonstrates the different measurements in the ascending aorta (1), false lumen at the entry (2) and descending aorta (3) in a 70 keV reconstruction.

Two regions of interest (ROIs) with a 1 cm^2^ area were placed in the ascending and descending aorta (true lumen) as well as the false lumen close to the entry; attenuation, as well as noise, (standard deviation of attenuation) were acquired at the energy levels of 40–190 keV in 10 keV increments. Additionally, two ROIs were placed in equivalent positions, as mentioned above in the true NC series and VNC series, both with the same slice thickness of 1 mm. The following Figure 1 exemplarily depicts the performed measurements at 70 keV in one slice.

The SNR was calculated by CT value divided by noise (standard deviation), as previously described [11].

### 2.4. Subjective Analysis

VME (40 keV, 70 keV, 110 keV, 150 keV and 190 keV), NC and VNC images were assessed in the previously mentioned reconstructions by two experienced radiologists (9 and 14 years of experiences in radiology) and by an experienced vascular and general surgeon (8 years of experience) on a five-point Likert scale which is pointed out in the following Table 1. Raters were allowed to change the window settings and were not blinded regarding the reconstruction they were currently assessing. To sum up the ratings, the scores were added together and means and standard deviations were calculated.

### 2.5. Statistical Analysis

Statistical analysis was performed with SPSS 29.0.1.0 (IBM, Armonk, NY, USA). Estimates were provided as averages standard deviation if normally distributed, median (quartile 1; quartile 3) or frequency (percentage).

Kolmogorov–Smirnov testing was used for normal distribution, the Wilcoxon-rank test was used to test for the correlation for non-normal distributed data.

A *p*-value equal/less than 0.003 was considered statistically significant, *p*-value was adjusted for multiple tests with the Bonferroni method.

## 3. Results

### 3.1. Baseline Study Characteristics

A total of 28 patients were included in this study, of which 8 (28.6%) were females and 20 (71.4%) males. Further details are shown in Table 2.

Out of these 28 patients, 12 (43%) showed a Stanford A dissection, 3 (25%) of them were already surgically treated, 27 of 28 (96%) showed a dissection membrane extending into the descending aorta and 9 (67%) of them were treated with an endovascular repair.

### 3.2. Objective Results

#### 3.2.1. Low-Energy Comparisons

##### Overall Data

The overall data (including all vascular segments) at 40 keV showed the highest values for vascular attenuation. At 40 keV, all measurements showed a median of 922 [780; 1127] HU, which decreased to 626 HU [536; 759] at 50 keV and further decreased with increasing energy levels.

Table 3 shows the median and interquartile ranges of the acquired attenuations of the overall data and specific vascular segments.

The SNR showed only a minor increase at lower energy levels from 40 keV (13 [9.7; 16.7]) to 70 keV (14 [10.8; 17]) but then reached 20 [15.6; 24.3] at 110 keV.

##### Vascular Segment-Specific Data

**Figure 2:**
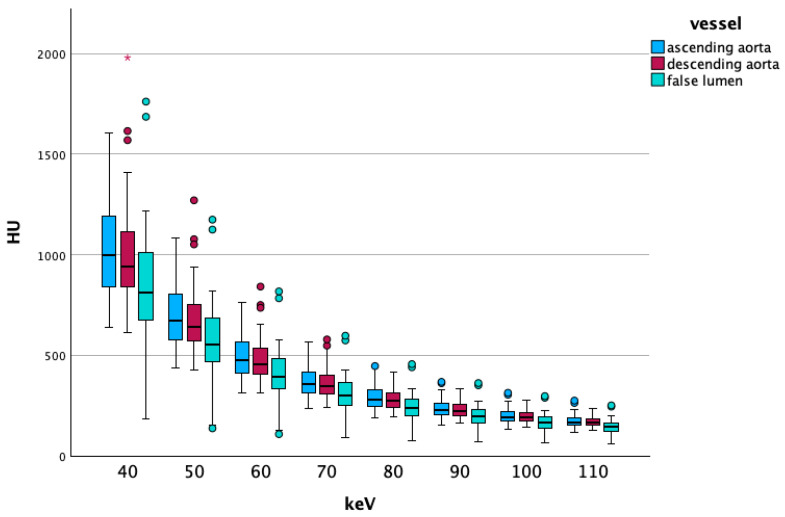
Shows a boxplot of the attenuation values of lower energy levels, comparing the ascending and descending aorta and the false lumen. Dots and asterisks represent outliers and extreme outliers.

Like in the overall data, the vascular segment-specific data also showed that vascular attenuation increased at lower energy levels. The ascending aorta showed higher CT values than the descending aorta, which in turn were higher than false lumen CT values. The difference in these changes increased with lower energy levels. Figure 2 visualizes these findings. With increasing energy levels, noise decreased, as the ascending part of the aorta showed the lowest noise values, followed by the descending part, and ultimately CT values of the false lumen of the dissection. The comparisons between the ascending and descending aorta showed significant differences in all comparisons below 90 keV, at 90 keV, we found a *p*-value of 0.013. Comparisons between the descending aorta and false lumen were significant on all energy levels from 40 to 190 keV (all *p*-values < 0.001). We saw the same results when comparing the ascending aorta with the false lumen, all comparisons showed highly significant differences (*p* < 0.001).

**Figure 3:**
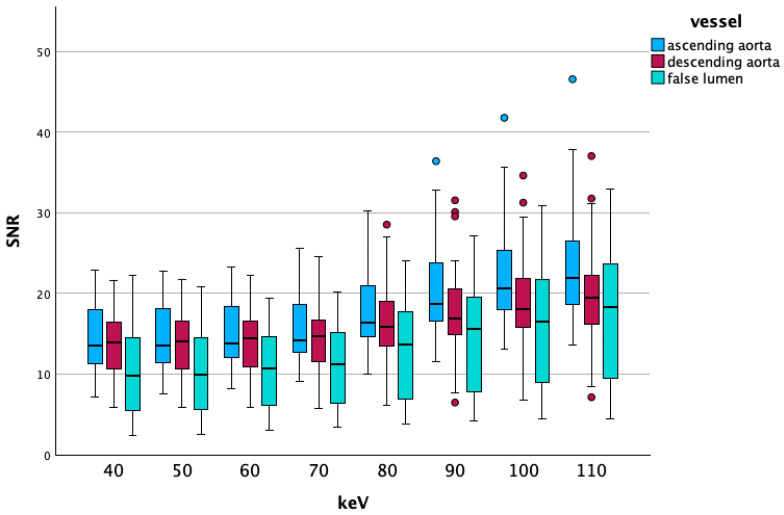
Visualizes the SNR changes on lower energy levels. The slight increase in the SNR on the lowest VME reconstructions, which was visible in the summed up vascular segments, is also present in vascular-specific data. The false lumen shows lower values compared to the ascending and descending aorta. Differences between the ascending and descending aorta become more evident at higher energy levels.

Figure 3 shows boxplots of the SNR on energy levels 40 to 110 keV from the different vascular segments, outliers are marked with dots.

#### 3.2.2. High-Energy/VNC/NC Comparisons

##### Overall Data

In our overall dataset, NC scans showed the lowest attenuation (48 [42; 55] HU), followed by VNC reconstructions (66 [52; 73] HU). The 190 keV reconstructions showed the lowest attenuation values of all VME reconstructions (97 [89; 105] HU) but these values were still above the VNC reconstructions, these comparisons all showed significant differences (*p* < 0.001). Comparing NC scans with VNC and higher keV VME reconstructions showed significant differences for all pairings (*p* < 0.001). The SNR showed the highest values at 120 keV (21 [15.8; 25.1]), with a continuous decrease up to 190 keV (16.5 [12.1; 21]). VNC reconstructions had a median SNR of 4.1 [3.4; 4.9], NC was even lower with 3.1 [2.6; 3.6].

##### Vascular Segment-Specific Data

**Figure 4:**
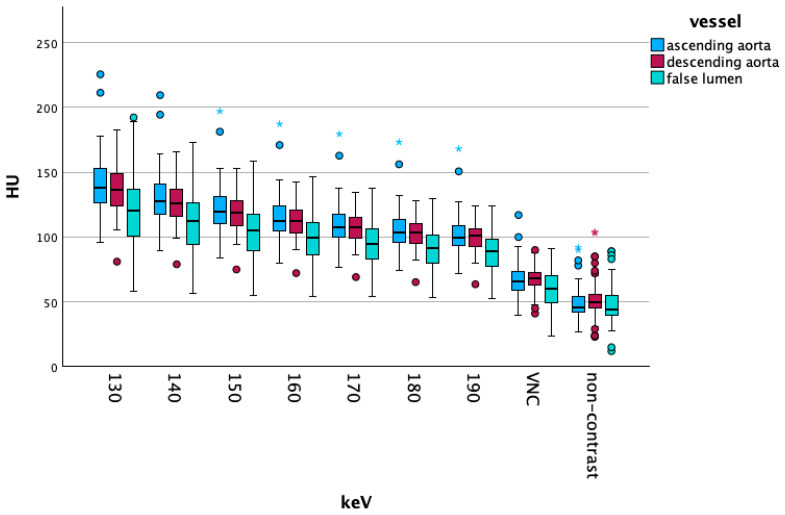
Demonstrates the attenuation for higher energy levels and compares those with virtual non-contrast (VNC) reconstructions and non-contrast scans. Dots and asterisks show outliers (dots) and extreme outliers (asterisks).

**Figure 5:**
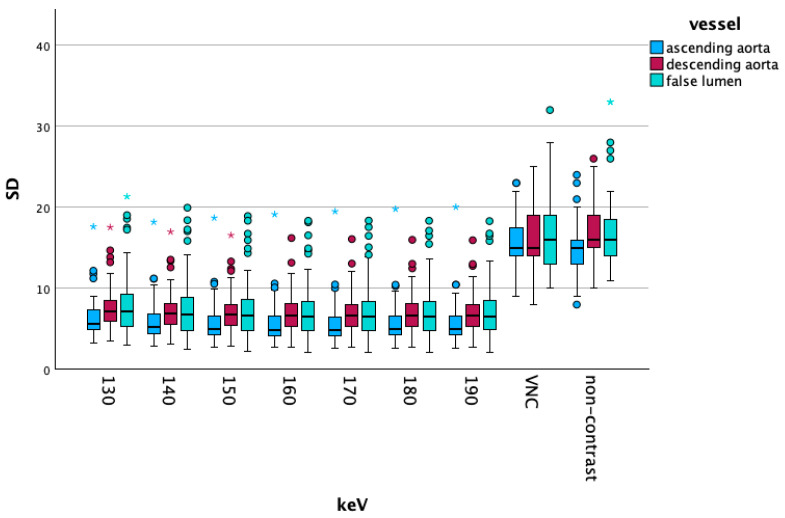
Shows standard deviation (SD) changes for higher energy levels and compares them with virtual non-contrast (VNC) reconstructions and non-contrast scans. Dots and asterisks show outliers (dots) and extreme outliers (asterisks).

Figure 4 shows the attenuation values of the high-energy VME, VNC and NC reconstructions of the three vascular segments. Figure 5 visualizes the corresponding standard deviation.

When comparing vascular segments with VNC data, we saw a significant difference in the comparison of the descending aorta with the false lumen (*p* = 0.002), as well as the ascending aorta with the false lumen (*p* < 0.001). The difference between the ascending and descending aorta showed no significant difference (*p* = 0.642).

The ascending vs. descending aorta on the NC scan also showed no significant differences (*p* = 0.013) and the descending aorta vs. the false lumen was significant (*p* = 0.003), whereas the ascending aorta vs. the false lumen showed no significant differences (0.913).

**Figure 6:**
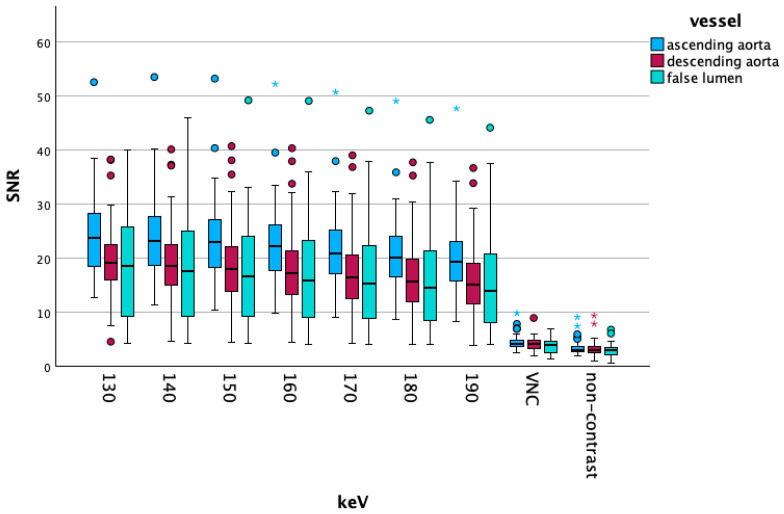
Demonstrates the changes in the SNR at higher energy levels and non-contrast as well as virtual non-contrast (VNC) reconstructions, outliers are marked with dots and asterisks.

The SNR in the different vascular segments is visualized in the following Figure 6. The SNR showed significant differences in all measured vessel segments between VNC and NC (*p* < 0.001).

### 3.3. Subjective Results

By summing up the subjective Likert ratings, we created the scores, which are presented in Table 4. Dissection assessability showed a Fleiss’ Kappa of 0.646, so a substantial inter-reader agreement and subjective non-contrast imaging showed a Fleiss’ Kappa of 0.34, so a fair agreement [15].

## 4. Discussion

This study focused on the evaluation of VME reconstructions derived from contrast-enhanced PCDCT in patients with AD, across different vascular segments. In addition, high-energy VME and VNC images were compared to the true NC phase to determine their potential as surrogates, particularly in the diagnostic workup of AD.

### 4.1. Low keV Data

As previously described, contrast media effects increase with lower energy reconstructions. VME images at 40 keV showed an overall 47% higher attenuation than the next energy level of 50 keV. At 40 keV, the attenuation in the ascending aorta was only 6% higher than in the descending aorta, but 23% greater than in the false lumen. The descending aorta demonstrated 16% higher attenuation values. At the energy level of 70 keV, CT values differed by 3%, when comparing the ascending and descending aorta, 20% compared with the false lumen and ascending aorta and 16% for the comparison between the descending aorta and false lumen. The SNR showed the highest values at 120 keV, 40 keV was 38% lower than the highest values with increasing SNR to 120 keV.

Most likely due to altered hemodynamics in AD patients, contrast media flow and dilution effects in the ascending aorta showed the highest attenuation values compared to descending aorta and the false lumen of the dissection. Changing contrast media effects through lowering the energy level were already described for VME imaging on dual energy CT and proved helpful, e.g., in identifying arterial bleedings [16] or endoleaks [17] and was also used for PCDCT in the differentiation of lung injuries and atelectasis [13] or visualization of bronchial arteries [18]. Also more recent studies have proven low-energy reconstructions to show sufficient subjective vascular imaging quality [19].

As we measured the false lumen’s attenuation CT values directly at the identified entry, it is remarkable that the false lumen still showed significantly lower attenuation values compared with the descending aorta (right before entry). By lowering the energy, these differences can be even more accentuated, which can be a helpful feature in identifying the correct and the false lumen of the AD. Also, TEM classification [4] requires the identification of the entry. With the above-mentioned dilution and hemodynamic effects, it seems logical, that from the dissection entry into the false lumen, attenuation decreases. Therefore, the CT values are—in early contrast media phases—the highest closer to the entry. Our study suggests that lower energy reconstructions might not only be helpful in identifying the false lumen, but also the entry, by finding areas with the highest CT values, especially in VME reconstructions close to the k-edge of iodine. Further studies in correlation with magnetic resonance imaging and the possible flow images [20,21] might offer further insights in contrast media dynamics and add to the understanding of blood flow in aortic dissection patients and the growth of false lumen and resulting risk stratification.

### 4.2. High keV and NC/VNC Scans

We found that true NC scans showed 28% lower attenuation relative to VNC reconstructions and up to 51% lower CT values compared to high-energy VME images at 190 keV, highlighting substantial differences relevant for imaging interpretation in ADs. For all vascular segments, the false lumen showed the lowest mean attenuation, in the NC series it was 4% lower than the ascending aorta, and 13% lower than in the descending part. In VNC reconstructions, the false lumen had 8% lower CT values than the ascending aorta and a value 11% lower than the descending aorta. The SNR was lowest in the NC series, significantly higher in the VNC series and then fourfold higher in VME reconstructions (190 keV), increasing up to 120 keV.

Risch et al. conducted a study using PCDCT and reported significant differences between true NC images and VNC reconstructions in aortic attenuation, which aligns with the findings of our study [14]. They did not take VME reconstructions into account and their research found lower attenuation values in VNC reconstructions compared to NC series in the aorta. Turrion Gomollon et al. examined endoleak detection on a PCDCT with a triphasic CT scan and two different readouts. They found that biphasic CT scans with virtual non-iodine imaging might include some erroneous calcium subtraction but could be a good alternative to triphasic CT scans in this indication group. They also did not include VME images on high-energy levels for contrast media removal, low-energy images for enhancing contrast media effects nor did they examine objective image parameters in their research [22]. Compared to these above-mentioned studies, our research did not include portal venous phase.

The results of the subjective imaging regarding the “non-contrast aspects” of the images in VNC reconstructions compared to NC images is in line with the available literature [23].

**Figure 7:**
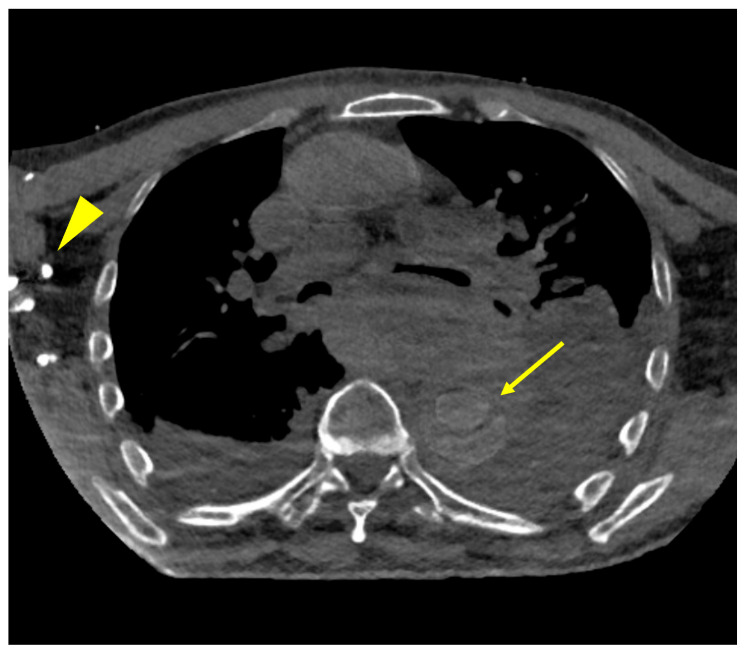
Shows a VNC reconstruction with contrast media visible on the right side (arrowhead) which was the injection arm, also in the descending aorta, minor contrast media effects can be seen with a slightly visible dissection membrane (arrow).

Several VNC reconstructions showed major contrast effects at the inflowing vein and still had minor visibility of the contrast medium, which is pictured in the following Figure 7.

It is surprising that we found significant differences in true NC scans for the descending aorta and false lumen. CT values for the descending aorta showed higher median values (50 HU) than the other vascular segments (46 and 44 HU) which might be due to the small study sample. The same effect can be seen in VNC reconstructions, in the contrast-enhanced series, the descending aorta had similar to slightly lower CT values than the ascending aorta (and higher than the false lumen in all cases).

Monoenergetic+ mode added to the overall image quality by calculating images from a subset of low noise and low keV assets, previous studies not using that calculation showed higher noise values especially for the low keV reconstructions [11]. This might be the reason for the lower noise values at higher energy levels in VME reconstructions compared to true NC and VNC images, also affecting the calculation of SNR as Monoenergetic+ denoises the VME reconstructions. Also, it seems that higher contrast medium concentrations might result in lower noise in Monoenergetic+ mode, which can be seen in higher CT values in the ascending aorta but lower standard deviations for lower energy levels.

Our study shows several limitations. First and overall, we only have a limited number of patients included. Also, our study population was very heterogenous with different types of dissection. These two important limitations can heavily influence confidence in non-significant and marginally significant findings. The heterogeneity of the study population with patients of treated and untreated pathologies could have had an impact on contrast medium dynamics as well. Pure Calcium VNC was not calculated in our research as previous studies showed almost no attenuation difference for arterial phase between these two VNC reconstructions [14].

Further studies should investigate the effect of different iterative reconstructions, which can also add to the image quality [24]. More research regarding the diagnostic accuracy of low-energy reconstructions in entry localization, including sensitivity and specificity, is needed to see if the above-mentioned effects are of clinical relevance.

As a conclusion, in AD patients, lowering the energy levels in VME reconstructions might be helpful in differing between the true and false lumen with an optimum at 40 keV, but regarding NC aspects, a true NC series cannot be replaced by a VNC series or even high-energy VME reconstructions.

## Figures and Tables

**Table 1:** Demonstrating the subjective ratings of dissection assessability and subjective native imaging on a five-point Likert scale.

Rating	Dissection Assessability	Subjective Non-Contrast Imaging
1	no dissection membrane/details visible	complete contrast media effect, vascular wall calcification might be obscured
2	slight visibility of the dissection membrane and other vascular details, no diagnostic quality	major contrast medium visibility, limited access to vascular wall structures including calcifications
3	clearly visible dissection membrane and other vascular details, major pathologies of the intima can be seen	average contrast media aspect, vessel walls and structures can be judged with an appropriate diagnostic quality
4	dissection membrane/details visible with minor impairment of diagnostic quality	minor contrast media effects, vascular wall and calcifications still fully visible
5	all dissection aspects and details, including entry can be clearly seen	no contrast media visible, vessel wall including calcification fully assessable

**Table 2:** Shows age and the dose parameters of the non-enhanced and the contrast-enhanced scans.

	Overall	Female	Male
Age	66 [52; 77]	67 [63; 78]	63 [49; 72]
CTDi non enhanced	7.23 ± 2.29	6.23 ± 2.05	7.69 ± 2.29
DLP non enhanced	480.46 ± 201.24	399.88 ± 149.35	512.70 ± 213.31
CTDi contrast	5.02 ± 1.77	4.49 ± 1.75	5.24 ± 1.78
DLP contrast	386.71 ± 144.97	338.88 ± 121.59	405.85 ± 151.87

**Table 3:** Shows the attenuation values of all measured data and the aortic segments and compares them on different energy levels and reconstructions, overall data represents all vascular segments together.

	Overall Data [HU]	Ascending Aorta [HU]	Descending Aorta [HU]	False Lumen [HU]
40 keV	922 [780; 1127]	997 [838; 1192]	940 [841; 1116]	810 [674; 1015]
50 keV	626 [536; 759]	674 [574; 802]	639 [572; 754]	552 [466; 686]
60 keV	445 [390; 536]	478 [412; 565]	457 [406; 536]	394 [334; 486]
70 keV	332 [297; 401]	357 [310; 419]	346 [305; 401]	298 [248; 363]
80 keV	261 [239; 313]	279 [246; 326]	273 [242; 314]	240 [197; 285]
90 keV	215 [196; 254]	227 [204; 264]	224 [201; 255]	196 [162; 230]
100 keV	184 [167; 212]	191 [174; 220]	191 [172; 214]	168 [138; 192]
110 keV	162 [145; 184]	167 [154; 190]	167 [151; 186]	145 [121; 166]
120 keV	146 [131; 163]	152 [137; 170]	149 [136; 165]	131 [111; 148]
130 keV	133 [120; 147]	138 [126; 155]	136 [124; 149]	121 [100; 137]
140 keV	123 [112; 135]	127 [117; 143]	126 [116; 137]	113 [94; 127]
150 keV	116 [105; 126]	120 [110; 133]	119 [108; 128]	105 [89; 119]
160 keV	110 [99; 113]	113 [105; 124]	112 [103; 121]	99 [85; 112]
170 keV	105 [95; 113]	108 [99; 118]	108 [99; 115]	95 [82; 106]
180 keV	100 [92; 109]	103 [96; 114]	104 [95; 110]	91 [79; 102]
190 keV	97 [89; 105]	100 [93; 109]	101 [92; 107]	89 [77; 99]
VNC	66 [57; 73]	66 [59; 74]	68 [63; 73]	61 [49; 71]
NC	48 [42; 55]	46 [42; 55]	50 [45; 57]	44 [39; 55]

**Table 4:** Shows the sums of the Likert ratings of all of the raters added up regarding dissection assessability and subjective native imaging on the different levels, a maximum of 420 could be reached. The values in parentheses represent the mean and the standard deviation of the ratings.

	Dissection Assessability	Subjective Non-Contrast Imaging
40 keV	420 (5.00 ± 0.00)	195 (2.33 ± 0.54)
70 keV	416 (4.95 ± 0.21)	192 (2.29 ± 0.77)
110 keV	251 (2.99 ± 0.59)	262 (3.12 ± 0.82)
150 keV	179 (2.13 ± 0.46)	317 (3.77 ± 0.53)
190 keV	161 (1.92 ± 0.42)	346 (4.12 ± 0.43)
VNC	129 (1.54 ± 0.50)	400 (4.76 ± 0.43)
NC	92 (1.10 ± 0.30)	420 (5.00 ± 0.00)

## Data Availability

Data are available from the authors on demand.

## Abbreviations

The following abbreviations are used in this manuscript:

CT	Computed tomography
AD	Aortic dissection
PCDCT	Photon-counting detector CT
VME	Virtual monoenergetic
VNC	Virtual non-contrast
NC	Non-contrast
SNR	Signal-to-noise ratio
CTDI	CT dose index
DLP	Dose length product
ROI	Region of interest
